# Magnetic hardening of Nd-Ce-Fe-B films with high Ce concentration

**DOI:** 10.1038/s41598-018-29907-4

**Published:** 2018-08-02

**Authors:** Hanyang Ren, Nadeem Abbas, Yang Liu, Hong Tang, Wanglin Gui, Jianzhong Ding, J. Ping Liu, Weixing Xia, Juan Du, Jian Zhang

**Affiliations:** 1 CAS Key Laboratory of Magnetic Materials and Devices, Ningbo Institute of Materials Technology and Engineering, Chinese Academy of Sciences, Ningbo, 315201 China; 2 Ningbo University, Ningbo, 315211 China; 3 Zhejiang Province Key Laboratory of Magnetic Materials and Application Technology, Ningbo Institute of Materials Technology and Engineering, Chinese Academy of Sciences, Ningbo, 315201 China; 4 Department of Physics, University of Texas at Arlington, Arlington, Texas 76019 USA

## Abstract

Partial substitution of Ce in Nd-Fe-B magnets is a feasible way to cope with the crisis of Nd and Dy in Nd-Fe-B production and reduce the cost of Nd-Fe-B magnets. In the present paper, the Nd-Ce-Fe-B films with high performance have been successfully fabricated by using an ultra-high vacuum (UHV) magnetron sputtering system. High magnetic performance with a ceorcivity of 13.3 kOe, a remanence of 11.4 kGs and a maximum energy product of 29.4 GMOe is obtained with the Ce substitution for more than 50 wt.% Nd without Dy addition. The high coercivity and (BH)_max_ achieved in this work are much larger than those of previously reported Nd-Ce-Fe-B magnets with the same Ce concentration. The phase structure, microstructure and coercivity mechanism are analyzed. The coercivity mechanism is determined to be mainly dominated by nucleation. Based on the microstructure observation and coercivity mechanism analysis, the fine and well separated grains, smooth grain surface, small and less inhomogeneities should be responsible for the high coercivity. Our results encourage the further improvement of magnetic properties in Ce magnets including the bulk material with high Ce concentration.

## Introduction

The Nd-Fe-B magnet, a key material related to the crucial energy efficiency technology^1^, has been widely used in different applications such as computer, aerospace, military, automotive industry, medical treatment and clean energy etc.^2,3^. Owning to the rapid rise of the requirement for Nd-Fe-B magnet, the Nd-Fe-B industry has a huge demand for rare-earth (RE) elements (Nd, Pr and Dy, etc.) every year. RE resources are divided into two types by supply risk versus importance to clear energy: less abundant and high abundant RE. The elements such as Neodymium (Nd) and Dysprosium (Dy), basic and important materials, are less abundant and more expensive^4^. On the other hand, the Ce element, which belongs to the second type, is more abundant and cheap. Ce is now a surplus resource because the RE elements are symbiotic. They need to go through separation and purification, which are produced together. In order to meet the challenge of Nd and Dy crisis in Nd-Fe-B production and utilize the abundant Ce resource and reduce the cost of Nd-Fe-B magnet, it is imperative to develop and study the Nd-Ce-Fe-B magnets in which a partial or total Nd is replaced by Ce. The addition of Ce in Nd-Fe-B magnets usually causes a big decline in performance, especially for high Ce concentration. So how to use Ce as a substitution with a slight change of property of permanent magnet is still an important issue. A lot of researchers have focused on the use of Ce replacing Nd in RE-Fe-B magnets. As reported earlier, using Ce as partial substitution for didymium (Pr-Nd alloy, abbreviated to Di) with 40 wt.% content, sintered Ce-Di-Fe-B magnets has achieved a maximum energy product (BH)_max_ of 28.2 MGOe and a coercivity of 9.2 kOe^5^. Better magnetic properties of sintered magnets have been reported for a Ce substitution of 30 wt.% with a (BH)_max_ of 43.3 MGOe and a coercivity of 9.26 kOe by using a double main phase alloy method^6^. This method has been proved to be a powerful approach for fabricating the high-performance Ce magnets. With 45 wt% Ce substitution for Di in [(Pr,Nd)_0.55_Ce_0.45_]_30.5_Fe_bal_M_1.0_B_1.0_ sintered magnet, good magnetic properties of B_r_ = 12.4 kG, H_cj_ = 9.0 kOe and (BH)_max_ = 36.7 MGOe are achieved^7^. The REFe_2_ phase is found to play a positive role on optimizing the microstructure in Ce magnets^7^. As for 50 wt.% Ce substitution, the properties get declined rapidly. A high coercivity of 7.7 kOe and a (BH)_max_ of 14.4MGOe are achieved for the Nd_6_Ce_6_Fe_82_B_6_ sample fabricated by a melt-spinning method^8^. The coercivity of 4.9 kOe is also obtained for (Nd_(1−x)_Ce_x_)_2_Fe_14_B melt spinning sample (x = 0.5) which is further enhanced to 8 kOe after Co addition. Due to a synergistic effect between Co and Ce, the Nd-Ce-Fe-Co-B rapidly solidified ribbon shows a better magnetic properties, especially at high temperatures^4,9^. The Ce substitution melt-spun powders have the potential of being a lower cost alternative for low- to moderate-temperature applications^10^. Yan *et al*. reported the higher substitution with 56 wt.% didymium replaced by Ce in Di-Fe-B sintered magnets. A high coercivity (11.33 kOe) is obtained. However, this high coercivity magnet has added 3 wt.% Dy^11,12^. For 60 wt.% Ce substitution, a high coercivity of 9 kOe is obtained in the ribbons, but their (BH)_max_ (about 9.2 MGOe) is low^13^. The cheap La and La-Ce alloy are also used to replace didymium in Nd-Fe-B sintered magnets. A high (BH)_max_ of 42.2 MGOe and a coercivity of 9–10 kOe are achieved in (Pr, Nd)_20.3_(La, Ce)_9.5_Gd_1.7_Fe_bal_M_1.1_B_1.0_ sintered magnet when the La-Ce substitution is as high as 36 wt.%. This magnet shows a good cost-performance in comparison with a 48.9 MGOe La/Ce-free commercial magnet^14^. It is also found that the La-Ce doping conduces to the magnetically favorable Ce^3+^ state^15^. For 50 wt.% La, Ce and La-Ce substitution (La/Ce ratio is 1.86), sintered magnets achieve the coercivity of 0.8 kOe, 5.7 kOe and 6.6 kOe, respectively^15^. With the variation of Ce, La and La-Ce substitution content, an abnormal increase of coercivity has been observed^9,11,13,16^. This phenomenon is more likely due to the variation in microstructure such as grain boundary instead of main phase, which is also supported by the micromagnetic simulation^17^. So it seems to be a feasible way to achieve the high-performance by partial substitution with a wide range of Ce. However, the fabrication of higher Ce concentration magnets with high performance is still a big challenge. And whether or not a higher performance can be achieved in the magnets added a large amount of Ce is still an important open question.

In comparison with the bulk sample, the advantage of the film is that it is easy to tailor the key microstructure parameters (the grain size, interface layer, etc.) that have major influences on the magnetic properties of permanent magnetic materials. The film system is best suited for a model system to study the optimum microstructure for the achievement of high performance in permanent magnetic materials^18–22^. In this paper, the Nd-Ce-Fe-B films are fabricated as a model system for investigating the high-content Ce substitution for Nd in Nd-Fe-B magnets and studying how the high performance, especially the high coercivity, can be obtained in the high Ce concentration magnets. A very large coercivity of about 13.3 kOe and a (BH)max of 29.4 MGOe are achieved for the film sample with a substitution of Ce for Nd of more than 50 wt.%. The coercivity is much higher than that of samples previously reported. Our results demonstrate that a large amount of Ce substitution in Nd-Fe-B magnet can achieve an excellent performance. The composition, phase structure, microstructure and coercivity mechanism of the film are investigated systematically. Understanding the origin of high-performance is very helpful for developing the bulk magnets with a high Ce concentration.

## Results

**Figure 1:**
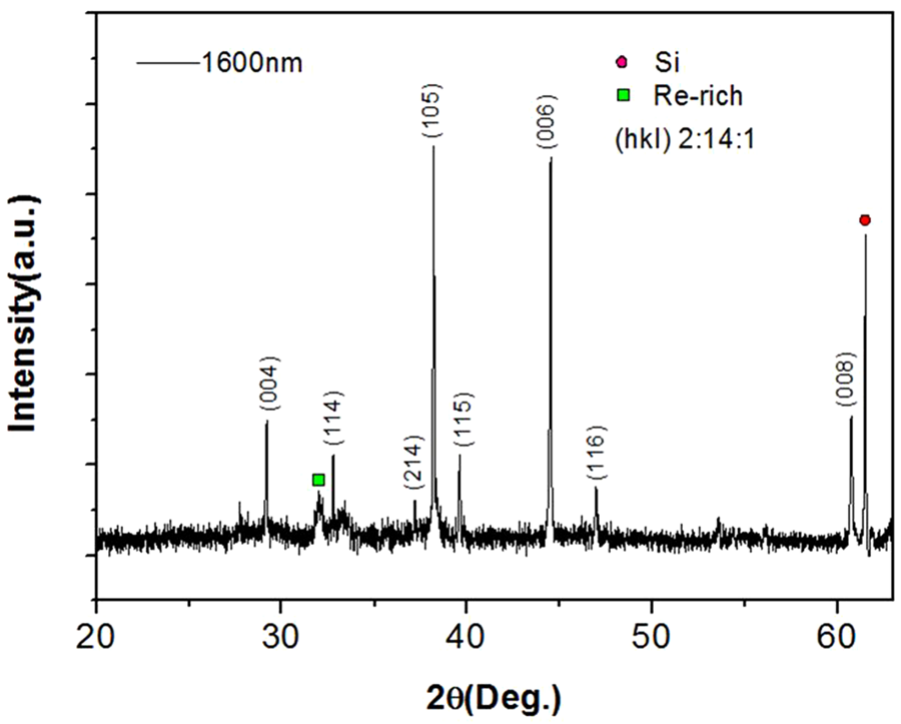
XRD pattern of 1600 nm thickness Nd-Ce-Fe-B film with nominal 50 wt.% Ce substitution.

Figure 1 shows the XRD pattern of the Nd-Ce-Fe-B film with 50 wt.% Ce substitution. The major peaks including (004), (114), (214), (105), (116), (006), (116) and (008) correspond to the Nd-Fe-B phase and are outlined in Fig. 1. The CeFe_2_ phase is not found from the XRD pattern.

It is well known that the magnetic properties of Nd-Ce-Fe-B magnets strongly depend on their Ce content and the addition of Ce will basically lead to a decline in coercivity and (BH)max. For the film fabrication, the change of RE content with the sputtering power has been reported in the Sm-Co based film^23^. In order to identify how much Ce concentration and how the change of RE content with the sputtering power for the Nd-Ce-Fe-B films, the compositions of the targets and films are analyzed carefully. The target and film with a nominal 30wt.% Ce substitution are firstly checked by ICP, an accurate technique usually used for the composition analysis. For performing the ICP measurement of the film, a special sample-preparation procedure has to be carried out as the following: the films are deposited for a very long time (7–10 hours) until a thick film about 7–10 μm is obtained. Such a thick film, which can easily peel off from the substrate, is utilized to perform the ICP measurement. Table 1 lists the composition of the target and films deposited with a power of 120 W and 200 W, respectively. It can be seen that the compound target has a similar composition with the nominal composition designed. For the film deposited with 120 W, the total RE content increases slightly as compared to the target composition. When the sputtering power rises to 200 W, however, the content of the RE elements in the films increases significantly compared to the target composition. The film deposited at 120 W has the similar Ce/Nd ratio with that of the target. However, the Ce/Nd ratio increases significantly from 120 W to 200 W.

**Table 1:** The composition of the nominal 30 wt.% Ce substitution Nd-Ce-Fe-B target and film measured by ICP.

	Nd(wt.%)	Ce(wt.%)	Fe(wt.%)	B(wt.%)	Total (wt.%)
Nominal composition	24.3	10.7	63.3	1.7	100
Target composition	23.25	8.84	66.45	1.46	100
Film composition (120 W sputtering power, ICP)	30	11.52	57.72	1.3	100
Film composition (200 W sputtering power, ICP)	39.68	31.48	28.21	0.63	100

**Figure 2:**
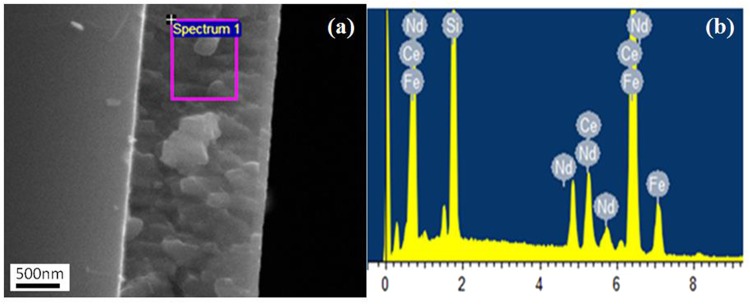
(**a**) Cross-sectional SEM image of 1600 nm thickness, nominal 50 wt.% Ce substitution Nd-Ce-Fe-B film; (**b**) EDX spectrum for the selected region shown in (**a**).

Table 2 shows the elemental composition of nominal 50 wt.% Ce substitution target and film measured by ICP and EDX (EDX can’t detect the content of B element), respectively. The target composition is very close to the nominal composition designed. The region is selected randomly for compositional analysis by EDX and is highlighted by a square in Fig. 2a. Figure 2b shows the EDS spectrum, confirming the presence of Nd, Ce and Fe elements. The Si peak in the spectrum is from the substrate. An increase of Ce content in the film is observed as compared to the target composition, which is consistent with the above results of the nominal 30 wt.% Ce substitution samples determined based on the ICP measurement shown in Table 1. The content of Ce increases from 13.7 to 17.1 and the content of Fe element has no significant change when depositing the Nd-Ce-Fe-B material from the target to the film. Ce accounts for 57 wt.% of the total RE content in this nominal 50 wt.% Ce substitution film.

**Table 2:** The composition of the nominal 50 wt.% Ce substitution Nd-Ce-Fe-B target and film measured by ICP and EDX.

	Nd (wt.%)	Ce (wt.%)	Fe (wt.%)	B (wt.%)	Total (wt.%)
Nominal composition	14.4	14.4	69.2	2	100
Target composition (ICP)	13.5	13.7	70.96	1.84	100
Film composition (140 W sputtering power, EDX)	13.13	17.1	69.77	/	100

**Figure 3:**
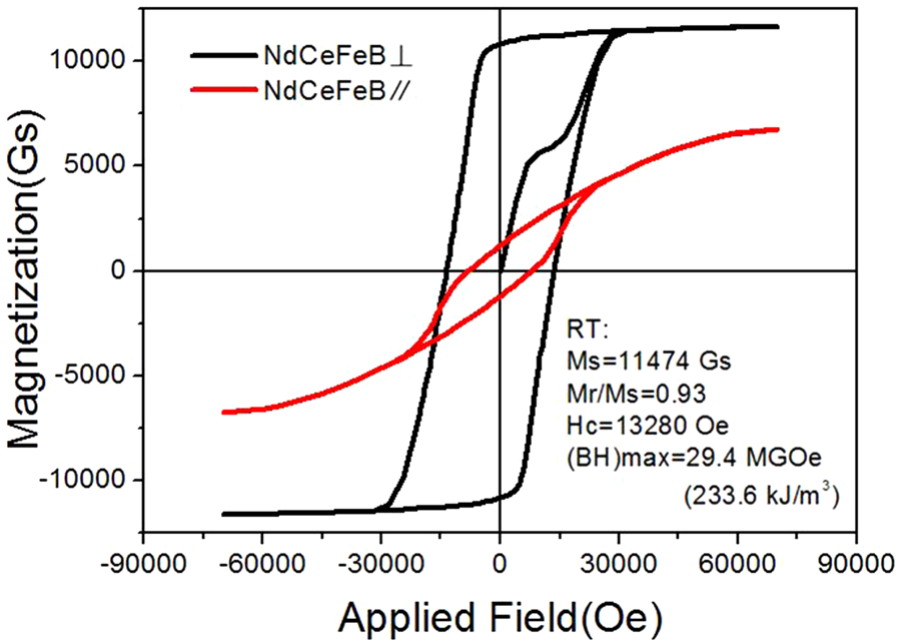
Hysteresis loops for the 1600 nm Nd-Ce-Fe-B film with nominal 50 wt.% Ce substitution measured along the out-of-plane (⊥) and in-plane (//) film directions.

Figure 3 presents the hysteresis loops of the Nd-Ce-Fe-B film with nominal 50 wt.% Ce substitution. The saturation magnetization, remanence ratio and coercivity are 11.5 kGs, 0.93 and 13.3 kOe, respectively. The maximum energy product (BH)_max_ is 29.4 MGOe (233.6 kJ/m^3^). There is a large difference for the hysteresis loops between measured along the out-of-plane and in-plane film directions, indicating that the film possesses a good magnetic anisotropy. The easy axis is along the out-of-plane of film surface and the hard axis is along the in-plane of film surface. The easy-axis demagnetization curve shows a good squareness.

**Figure 4:**
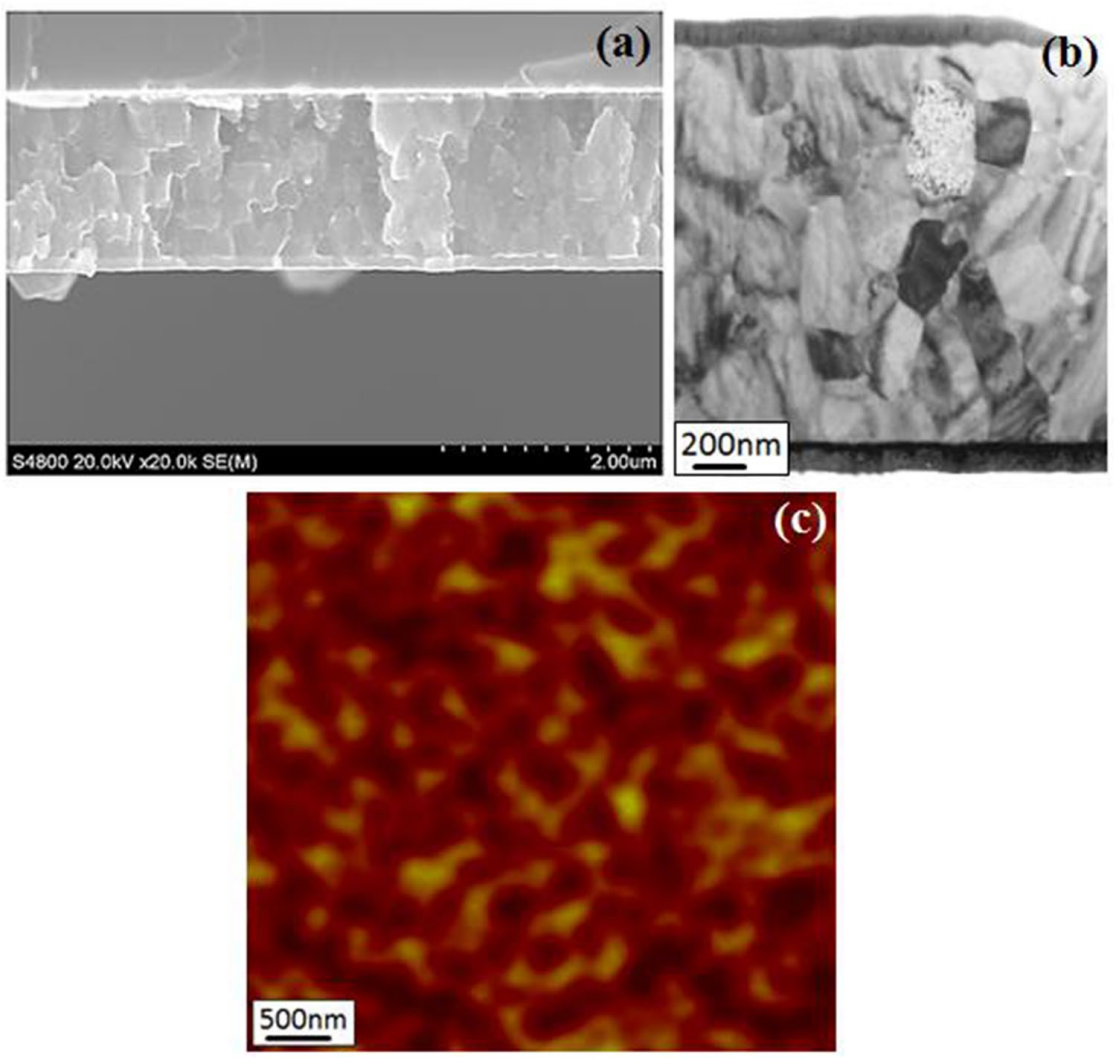
(**a**) Cross-sectional SEM image, (**b**) cross-sectional TEM image, and (**c**) MFM image on surface of 1600 nm thickness, nominal 50 wt.% Ce substitution Nd-Ce-Fe-B film.

The magnetic properties of permanent magnetic materials are basically determined by their phase structure as well as microstructure. The phase structure of Nd-Ce-Fe-B film is analyzed by XRD shown in Fig. 1. The microstructure of the Nd-Ce-Fe-B film is characterized by using both SEM and TEM. Figure 4a and b are cross-sectional SEM and TEM images of the 1600 nm thick film with nominal Ce substitution for 50 wt.% Nd, respectively. It can be seen from Fig. 4a that the trilayer film with a flat combination and uniform thickness.is tightly filled by grains and does not have any obvious interspace or hole, indicating a high quality of film. The TEM image in Fig. 4b shows the same trilayer structure and confirms the SEM results above. In addition, the average grain size determined from the SEM and TEM is similar (about 300 nm). It can also be observed from the TEM image that most of the grains are separated from each other by the boundary phase which should be the RE-rich phase^24^. Figure 4c shows a MFM image taken on the film surface. The MFM tip used here is only sensitive to the magnetic vector perpendicular to the film surface. The dark regions indicate the magnetic domain orientation perpendicular to the film surface. The bright regions show also the domain orientation perpendicular to the film surface but an adverse direction with the dark regions. The size of most magnetic domains is smaller than 500 nm.

**Figure 5:**
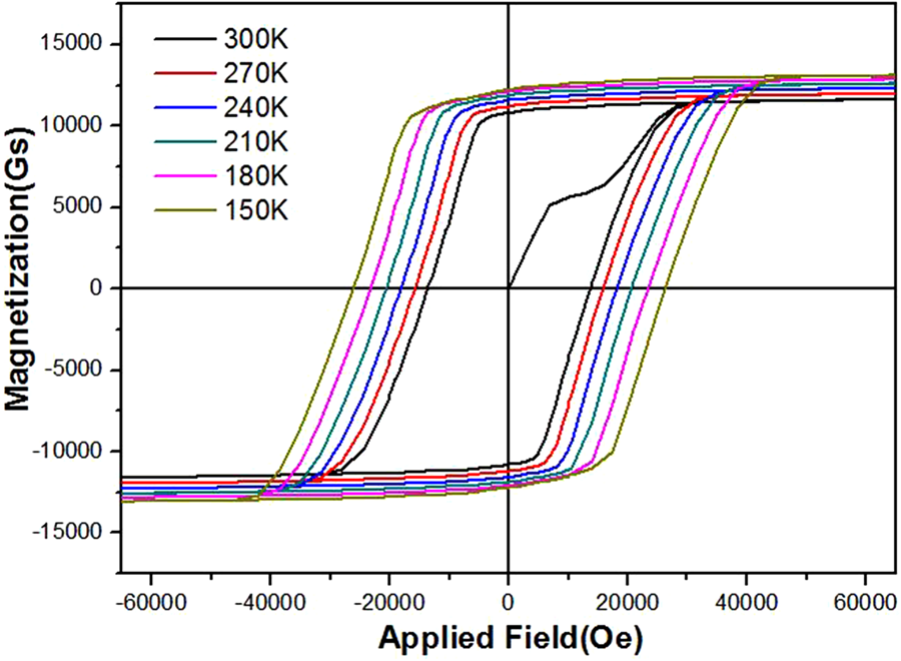
Hysteresis loops for nominal 50 wt.% Ce substituted Nd-Ce-Fe-B films at different temperatures.

**Figure 6:**
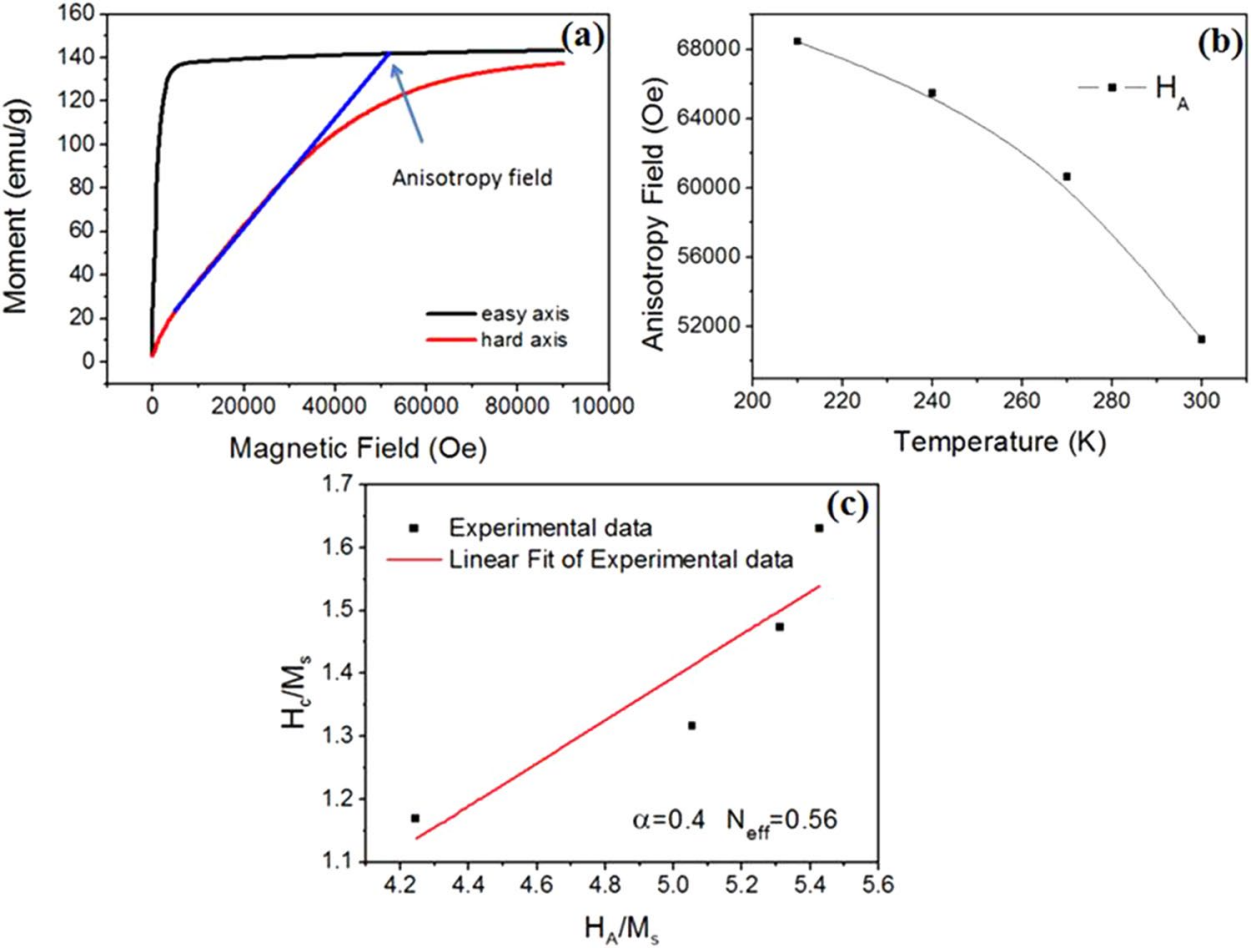
(**a**) Easy- and hard-axis magnetization curves for nominal 50 wt.% Ce substituted Nd-Ce-Fe-B alloy measured at room temperature; (**b**) The dependence of anisotropy field H_A_ on temperature for nominal 50 wt.% Ce substituted Nd-Ce-Fe-B alloy; (**c**) The dependence of H_c_/M_s_ on H_A_/M_s_ for nominal 50 wt.% Ce substituted Nd-Ce-Fe-B film.

The magnetic properties of permanent magnets are sensitive to temperature^9,25^. The temperature dependence of the hysteresis loops for the 50 wt.% Ce substitution Nd-Ce-Fe-B film has been measured at the temperature range from 150 K-300 K, which is shown in Fig. 5. The coercivity increases from 13.3 kOe to 26.3 kOe with decreasing the temperature from 300 K to 150 K and the same is the trend for the saturation magnetization (from 11.5 kGs to12.6 kGs). In order to gain insight into the mechanism of high coercivity achieved in the high Ce content film, the temperature dependence of coercivity is derived from Fig. 5 and the coercivity mechanism is analyzed based on the micro-magnetic model. A formula showing the relationship between coercivity, anisotropy field and saturation magnetization is given as follows^26^:

1 $${H}_{c}({\rm{T}})=\alpha {H}_{A}({\rm{T}})-{N}_{eff}{M}_{s}(T)$$

where ${H}_{c}$ and ${M}_{s}\,\,$are the coercivity and saturation magnetization, respectively. *H*_*A*_ is the intrinsically magnetic anisotropy field, which is obtained from the easy and hard axis magnetization curves^27,28^ as shown in Fig. 6a. The anisotropy fields of 50 wt.% substituted Nd-Ce-Fe-B at different temperatures (210 K–300 K) are attained by the same method shown in Fig. 6a. The dependence of anisotropy field on the temperature is shown in Fig. 6b. It can be seen that the anisotropy field has a quick drop with the increase of temperature. The parameter ${\rm{\alpha }}$ describes the reduction in the anisotropy field owing to crystallographic defects on grain surface and grain misalignment. ${N}_{eff}$ is the demagnetization factor which is related to the local demagnetization field. Figure 6c shows the dependence of H_c_/M_s_ on H_A_/M_s_ for 50 wt.% Ce substituted Nd-Ce-Fe-B film. The values of ${\rm{\alpha }}$ and ${N}_{eff}$ can be obtained by using a linear fit, and they are 0.4 and 0.56, respectively.

## Discussion

In this work, we successfully prepared the high-performance Nd-Ce-Fe-B film with a high Ce concentration of more than 50 wt.% Ce substitution. Although important progresses have been made^6–9,11,13–15^, the magnetic properties of Ce magnets with a Ce-substitution proportion higher than 50 wt.% are still low. The magnetic properties achieved in our film (see Fig. 3) are much higher than those of previously reported Nd-Ce-Fe-B magnets with same Ce concentration fabricated by other methods^4,8–10,13,15^, indicating the film fabrication with magnetron sputtering is a powerful way for the achievement of high-performance in permanent magnetic materials^18–22^. A large coercivity of 13.3 kOe and good squareness in hysteresis loop (Fig. 3) imply that the film may have a homogenous grain size distribution and good texture, which are consistent with the results of the XRD, SEM and TEM measurements (Figs 1, 2 and 4). For Nd-Ce-Fe-B magnets with a Ce substitution more than 50 wt.%, the coercivities of about 4.9–9 kOe have been reported^4,8–10,13,15^. The (BH)_max_ value 29.4 MGOe for our Nd-Ce-Fe-B film shown in Fig. 3 is reliable. The coercivity and (BH)_max_ values obtained in this work are much higher than what has been reported previously for the Nd-Ce-Fe-B magnets with same Ce concentration^4,8–10,13,15^.

It is necessary to ascertain whether or not such a high Ce substitution as designed is really realized in the present film since the magnetic properties of Ce magnets depend on the Ce content. The compositions of the target and film are analyzed by both ICP and EDX. The preparation of the film sample for ICP analysis is not easy, but it can give more reliable data. We find a way to do the accurate ICP analysis on the film sample. The point is that the thick Nd-Ce-Fe-B film can peel off from the substrate. The composition-analysis results shown in Tables 1 and 2 confirm that the Ce content in the films fabricated from 120 W to 200 W is higher than the nominal composition designed. Using of high sputtering power (120 W–200 W) for fabricating the films guarantees the high Ce content. Our results give the definite evidences that the excellent magnetic behaviors can be really achieved for Nd-Ce-Fe-B magnet even for Ce substitution more than 50 wt.%. It demonstrates that the high-performance magnets can be obtained by replacing less abundant and expensive RE element with a more abundant and cheaper Ce element. Another notable result is the RE content rising with the sputter power in Nd-Ce-Fe-B film, which is totally different from the finding in Sm-Co-based film where the Sm content decreases with the sputter power^23^. Our result may provide the useful information for controlling the RE composition in Nd-Ce-Fe-B film.

The magnetic properties of permanent magnetic materials are basically determined by their phase structure as well as microstructure. The phase structure of Nd-Ce-Fe-B film is checked by XRD. The strong XRD peaks, i.e., (004), (105), (006) and (008), shown in Fig. 1 clearly indicate that the Nd-Ce-Fe-B grains in the film are oriented along the c axis with a well-texture perpendicular to the film surface. This is also consistent with the hysteresis-loop measurement shown in Fig. 3. The grain size observed from SEM and TEM images is about 300 nm. This size is close to the single domain size of Nd-Fe-B particle^29^, which should contribute to the high coercivity. The formation of boundary phase between adjacent grains has been observed from the TEM image. The boundary phase has been expected to cause a reduction in intergranular exchange coupling, which plays an important role in improving the coercivity^17,30^. The reduced intergranular exchange coupling is examined by the observation of magnetic domain structure with MFM (Fig. 4c). The MFM image shows that the magnetization direction of most domains is along the normal direction of the film surface, which is in agreement with the result shown in Fig. 3. Most domain sizes are smaller than 500 nm, close to the grain size (300 nm) determined from SEM and TEM. In a strongly magnetic interaction system, the domain size, which is much larger than the grain size, has been evidenced in previous studies^31,32^. Moreover, a deemed interaction-domain with a size (about few microns) much larger than the grain size of about 300 nm has been also observed by MFM in a hot deformed Nd-Fe-B magnet^33^. Our film shows a fine domain structure with a domain size closer to the grain size, demonstrating that the grains in Nd-Ce-Fe-B films are magnetically better separated from each other by the boundary phase. However, there are still some connection between “different” domains with same orientation, indicating a certain extent of magnetic coupling still exists between grains. This is possibly due to the boundary phase not continuously surrounding the grain, or the dipole interaction effect. Anyhow, the fine sized (300 nm) and better separated grains evidenced from SEM, TEM and MFM contribute to the high coercivity in the Nd-Ce-Fe-B film.

The rise of coercivity with decreasing temperature (Fig. 5) should be attributed to the enhancement of the anisotropy field (Fig. 6b). The coercivity mechanism is further analyzed based on the micro-magnetic model. The microstructure parameter ${\rm{\alpha }}$, actually, is given by ${{\rm{\alpha }}}_{K}{\alpha }_{\phi }$, where ${{\rm{\alpha }}}_{K}$ is the reduction in the anisotropy field owing to crystallographic defects on grain surface and ${\alpha }_{\phi }$ describes the grain misalignment. Here ${\alpha }_{\phi }$ is treated as a temperature independence parameter since it does not change so much for Nd-Fe-B at the temperature range of 210–300 K^34^ and also the Nd-Ce-Fe-B film has a good texture. Thereby a line fitting is used to determine the microstructure parameter α and ${N}_{eff}$ (see Fig. 6c). The obtained value of α is 0.4. Since ${\alpha }_{\phi }$ is lower than 1^34,35^, ${\alpha }_{K}\,\,$should be much larger than 0.3, strongly supporting that the nucleation is the leading mechanism for the coercivity of 50 wt.% Ce substituted Nd-Ce-Fe-B film^36^. However, the initial curve in Figs 3 and 5 shows a domain wall pinning character. This contradiction can basically be solved based on the fact that the magnetization reversal of well isolated hard grains with a single domain size, which should be controlled by the nucleation of reversal domains, exhibits also a pinning-like initial magnetization curve^37^. Since the grain size in the Nd-Ce-Fe-B film is close to the single domain size, it is highly likely that some single-domain sized and completely isolated grains exist, leading to the pinning-like initial magnetization curve. The α value of 0.4 is also higher than that of the Nd-Fe-B single layer film previously reported^19^. The high ${\rm{\alpha }}$ value of 0.4 indicates that the grains in Nd-Ce-Fe-B film have less misalignments and smooth surface with less defects, which can reduces the local reduction in magneto-crystalline anisotropy. The value of ${N}_{eff}$ for the 50 wt.% Ce substituted Nd-Ce-Fe-B film is about 0.56. It is worth noting that this value is much lower than that of sintered magnets reported^34,35^. ${N}_{eff}$ is related to the demagnetization field (stray field) which basically has various sources^38^: (a) macroscopic field from the sample surface charge, (b) structural stray field due to the nonmagnetic or weak-magnetic inhomogeneities including hole, defect, triangle grain boundary phase, precipitate, etc., (c) stray field of ferromagnetic grain itself, (d) misaligned grains. For both the film and sintered magnets, (a), (c) and (d) should be similar. Therefore, the large discrepancy in ${N}_{eff}$ should be mainly due to the difference in the structural stray field from nonmagnetic inhomogeneities. This is supported from the microstructural observation (Fig. 6a,b), which shows a very compact grain arrangement in Nd-Ce-Fe-B film. If we compare the microstructure of film shown in Fig. 4a,b with the typical microstructure of sintered magnets^29,39^, the remarkable differences in microstructure features between the film and sintered magnet are the sizes of grain and inhomogeneities such as Nd-rich phase at triple junction of grain boundary. The dramatical decrease in these sizes can reduce largely the local stray fields in the film^40,41^, leading to a low ${N}_{eff}$. The reasons why the decrease of grain size can bring about the reduction of local stray fields can be understood based on the following: (1) The local stray field can be reduced due to a low magnetic volume of reversed and size-decreased grain^41^; (2) The density of defects on the grain surface decrease with grain size^42–45^. The decreased numbers of surface defects cause the reduction of local stray fields. The bulk Ce sintered magnet has become a commercial product and it has the important applications. Our result suggests that the coercivity of sintered magnets can be further improved by reducing ${N}_{eff}$ through decreasing the size and number of inhomogeneities or size of grains. Anyhow, the fitting result about the values of ${\rm{\alpha }}$ and ${N}_{eff}$ based on the micromagnetic model provide a deep understanding on the coercivity mechanism for the high-performance Nd-Ce-Fe-B film. It also gives new clues for designing the high-performance Ce sintered magnet.

## Conclusions

In summary, high-performance Nd-Ce-Fe-B films are successfully fabricated by UHV magnetron sputtering. A high coercivity of 13.3 kOe, remanence ratio of 0.93, and maximum energy product (BH)_max_ of 29.4 MGOe (233.6 kJ/m^3^) are achieved for the Nd-Ce-Fe-B film with a substitution of Ce for Nd more than 50 wt.%. The compositional analysis shows that the RE content rises with the sputtering power. It also confirms that the substitution proportion of Ce for Nd in the film is no less than the nominal composition designed. The SEM, TEM and MFM images manifest that the Nd-Ce-Fe-B gains are fine with size about 300 nm and magnetically well-separated by RE-rich phase along the grain boundaries. A micro-magnetic model is utilized to gain insight into the coercivity mechanism. The coercivity of the Nd-Ce-Fe-B film is mainly controlled by nucleation mechanism. The fitting result gives the high value of α, indicating that the grain has a smooth surface and less misalignments causing an alleviation of local reduction in anisotropy field. The fitting value of *N*_*eff*_ for the film is much larger than that for sintered magnets, which is owing to the dramatically decreased sizes of grain and inhomogeneitis such as Nd-rich phase at triple junction of grain boundary. Our result demonstrates that the substitution of more abundant and cheaper Ce element for Nd with a high amount (more than 50 wt.%) can achieve the high performance. Our study is also of significance for understanding the mechanism of high-performance film/bulk magnets with high Ce concentration. It also provides the new clues for further improving the magnetic properties of Ce sintered magnet.

## Method

The Ta (100 nm)/Nd-Ce-Fe-B (1600 nm)/Ta (50 nm) films were deposited on Si (100) substrates by using an ultra-high vacuum (UHV) magnetron sputtering system. The base pressure of the chamber was better than 1 × 10^−6^ Pa. The argon pressure during sputtering was 0.8 Pa. A commercial Ta target of better than 99.9% purity was used for depositing an underlayer and a coverlayer for protection. The deposition temperature and sputtering power of underlayer and coverlayer were 300 °C and 120 W, respectively. The compound targets of Nd_24.3_Ce_10.7_Fe_63.3_B_1.7_ and Nd_14.4_Ce_14.4_Fe_69.2_B_2_ were used to deposit the Nd-Ce-Fe-B layer. The deposition temperature was 500 °C and sputtering power was from 120 W to 200 W. After deposition, the whole film was post-annealed at 720 °C for 3 min. The Nd_14.2_Ce_13.8_Fe_69.8_B_2.1_ alloy ingot was fabricated by arc-melting, and then annealed at 1050 °C for 5 days. A cylindrical shaped sample with size of Ф3*3 mm, prepared by mixing the alloy powder with epoxy resin and then aligning at a magnetic field of about 28 kOe, was used to measure the intrinsic properties of Nd-Ce-Fe-B alloy. The magnetic properties of the film with a size of about 5 mm × 2.8 mm were measured by a magnetic property measurement system (MPMS (SQUID) VSM) with a magnetic field up to 70 kOe. The surface area of the film was further accurately calculated by taking a photo and putting it in a Photoshop software. The microstructures were analyzed by scanning electronic microscopy (SEM) and transmission electron microscope (TEM). The magnetic domain structure was observed with magnetic force microscopy (MFM). The crystal structure and texture were determined by X-ray diffraction (XRD) with Cu Kα radiation. The compositions of targets and films were analyzed by inductive coupled plasma emission spectrometer (ICP) and energy dispersive X-ray spectrometric microanalysis (EDX).
